# Impact of Shortened Crop Rotation of Oilseed Rape on Soil and Rhizosphere Microbial Diversity in Relation to Yield Decline

**DOI:** 10.1371/journal.pone.0059859

**Published:** 2013-04-01

**Authors:** Sally Hilton, Amanda J. Bennett, Gary Keane, Gary D. Bending, David Chandler, Ron Stobart, Peter Mills

**Affiliations:** 1 School of Life Sciences, University of Warwick, Wellesbourne, Warwickshire, United Kingdom; 2 The National Institute of Agricultural Botany, Wymondham, Norfolk, United Kingdom; Wageningen University and Research Centre, The Netherlands

## Abstract

Oilseed rape (OSR) grown in monoculture shows a decline in yield relative to virgin OSR of up to 25%, but the mechanisms responsible are unknown. A long term field experiment of OSR grown in a range of rotations with wheat was used to determine whether shifts in fungal and bacterial populations of the rhizosphere and bulk soil were associated with the development of OSR yield decline. The communities of fungi and bacteria in the rhizosphere and bulk soil from the field experiment were profiled using terminal restriction fragment length polymorphism (TRFLP) and sequencing of cloned internal transcribed spacer regions and 16S rRNA genes, respectively. OSR cropping frequency had no effect on rhizosphere bacterial communities. However, the rhizosphere fungal communities from continuously grown OSR were significantly different to those from other rotations. This was due primarily to an increase in abundance of two fungi which showed 100% and 95% DNA identity to the plant pathogens *Olpidium brassicae* and *Pyrenochaeta lycopersici*, respectively. Real-time PCR confirmed that there was significantly more of these fungi in the continuously grown OSR than the other rotations. These two fungi were isolated from the field and used to inoculate OSR and *Brassica oleracea* grown under controlled conditions in a glasshouse to determine their effect on yield. At high doses, *Olpidium brassicae* reduced top growth and root biomass in seedlings and reduced branching and subsequent pod and seed production. *Pyrenochaeta* sp. formed lesions on the roots of seedlings, and at high doses delayed flowering and had a negative impact on seed quantity and quality.

## Introduction

Farmers have traditionally used crop rotation as a method of managing the productivity of the soil and reducing the effects of crop pests and diseases. Often these rotations involved growing four or five different crops in a sequence. However, with the intensification of agricultural production in recent decades, many farmers now specialise in just one or two crop species, using inputs of fertilizers and pesticides to compensate for the lack of rotation [1], [2]. This approach is not always successful, and declines in yield have been reported for a number of crops grown associated with continuous cropping or short rotation, including maize, soybean, wheat, potatoes, sugarcane and oilseed rape (OSR) [3]–[10]. Yield decline in OSR is a particular concern because of rising demand for the crop as a source of biofuel in addition to its cultivation for cooking oil and as an animal feed. OSR is also a very important part of the arable rotation and remains highly profitable when grown as a break crop for cereals. The global demand for biofuel has resulted in a rapid growth in world production of OSR, with 31 million hectares of land under cultivation in 2009, a 79% increase from 1989 [11]. In the UK, the average area used for growing OSR between 2001 and 2011 increased by 56% [12]. This increased demand for OSR has resulted in a higher proportion of OSR in rotations, with standard crop rotations of at least 1 in 5 being shortened by many farmers to 1 in 2. This has resulted in reports of yield decline, which vary from 3–12% in short rotation [8] to 25% in continuous cropping in the field trial used for this study [13]. The causes of yield decline are thought to be complex; various factors have been implicated for a range of crop species, including abiotic factors such as nutrient availability and land management practices, as well as biotic factors such as plant pathogens, autotoxicity of the crop, and changes in the soil or rhizosphere microbial community structure, which can subsequently have a deleterious effect on the crop [2], [9], [10]. The causal mechanisms for yield decline in OSR are not fully understood [9], [10], which is of significant concern given the importance of this crop to the agricultural economy.

In this paper, we investigated the relationship between OSR yield decline and changes in soil/rhizosphere microbial communities. Previous studies have established that plants influence microbial communities through root exudates and residues in the soil and there is evidence of an association between crop type and microbial community composition, including wheat, potato, strawberry and OSR [14], [15]. Furthermore, growing a crop continuously or in short rotation has been associated with a decline in the diversity of its rhizosphere microbial community [16]–[21]. It is possible that crop rotation provides a greater concentration and diversity of organic materials which may lead to a greater diversity of microbial communities. Yet, relatively little is known about the precise nature of these changes and their effects.

Some soil-borne microorganisms can be beneficial to the plant through the suppression of pathogens or by the production of plant growth-promoting substances, potentially resulting in increased vigour and a subsequent increase in yield [2], [22]. However, soil microorganisms may also include plant pathogens or deleterious rhizosphere microorganisms (DRMO), which may have a negative impact on plant growth and yield. Major plant pathogens typically cause obvious symptoms of disease, and can usually be controlled through chemical, biological or cultural means. However, the impact of DRMO on yield decline is difficult to quantify as these organisms usually restrict plant growth without any obvious symptoms [22].

This investigation aimed to relate soil/rhizosphere microbial community to yield decline in OSR. Our central hypothesis was that growing OSR continuously or in shortened rotation causes the build-up of populations of specific plant pathogens or DRMO with a concomitant effect on crop yield. The aims of this study were to: (i) determine fungal and bacterial communities associated with OSR grown in rotations of different frequencies of cropping with wheat, where yield decline has been observed; (ii) identify microorganisms that became more prominent with frequent cropping of OSR; (iii) determine if these prominent microorganisms impact on OSR yield using experiments under controlled conditions.

## Materials and Methods

### Ethics Statement

No specific permits were required for the described field studies. Permission to perform the field studies were granted by the landowner (The Morley Agricultural Foundation).

### Field Plot Experimental Design and Sampling Strategy

An established field trial based in East Anglia, UK (52° 33′ N and 1° 2′ E), investigating the effect of different frequencies of cropping of OSR (cv. Winner) and winter wheat (cv. Brompton) on OSR yield, was used to provide samples for this project via NIAB TAG and funded by HGCA (Project RD-2003-2922). This field trial showed a yield reduction in the continuous OSR (grown for four consecutive years) of 25% compared with virgin OSR [13] (Fig.S1).

The soil type was a sandy clay loam with a pH of 6.6 and available P, K, Mg and SO_4_ of 32.4, 111, 28 and 30.6 mg kg^−1^ respectively. The entire trial area was ploughed and pressed each season ahead of establishment. While specific drilling dates varied according to season, oilseed rape was typically drilled in early September, first winter wheat in the second half of September and subsequent wheat in mid-October [13]. All inputs were consistent with local commercial best practice, although prophylactic approaches have been used when necessary [23]. Oilseed rape treatments included applications of nitrogen (N) and sulphur (S) of 200 kg ha^−1^ N and 30 kg ha^−1^ S [23]. First wheat and subsequent wheat treatments (second wheat and later) received different input programmes but again approaches were typical of local best practice [23].

The field trial was in its fourth year when samples were collected in June 2007; four weeks prior to harvest. Five rotations were used in this study, which are shown in Table 1. Rotations were planted in randomised plots of 24×6 m and each rotation was replicated four times [13]. Each plot was further divided into three equal sub-plots longitudinally. The central sub-plot was used for yield data and the outer two sub-plots were used for destructive sampling.

**Table 1:** Cropping history of rotations sampled.

	Year of trial			
Rotation	1	2	3	4
Continuous OSR	O	O	O	O
‘Continuous’ wheat	O	W	W	W
OSR 1 in 2	W	O	W	O
OSR 1 in 3	O	W	W	O
Virgin OSR	W	W	W	O

Rhizosphere and bulk soil samples were collected in the 4^th^ year of the trial (O = OSR, W = wheat).

Bulk soil and rhizosphere samples were collected from the sub-plots of each of the four replicates of the five selected rotation treatments. For each replicate, three plants were excavated from the two sub-plots at approximately 6, 12 and 18 m along the length of the plot (six plants in total per replicate) and pooled. Bulk soil samples were collected at the same intervals, using a 30 cm auger (six samples pooled per replicate). Plants and bulk soil samples were taken back to the laboratory for processing. Roots were shaken free of loose soil and fine roots were cut into approximately 5 mm sections. Fine roots plus closely adhering soil were designated as the rhizosphere and sub-samples (0.5 g) of rhizosphere material were frozen for later molecular analyses. Bulk soil samples were sieved using a 3 mm sieve and sub-samples (0.5 g) were also frozen for molecular analyses.

### DNA Extraction and TRFLP Analysis

DNA was extracted from 0.5 g of each bulk soil and rhizosphere sample using the FastDNA® SPIN Kit for Soil (MP Biomedicals LLC, UK), according to the manufacturer’s instructions, with the exception that samples were homogenized in a Mini Beadbeater-8 cell disrupter for 3 minutes (Biospec products, Inc., USA) rather than a Fast-Prep machine. DNA samples were amplified with PCR primers universal to the internal transcribed spacer (ITS) region of fungi or the 16S rRNA gene of bacteria. The PCR master mix contained 47 µl Megamix (Microzone Limited, UK) and 10 ng DNA along with 1 µl of a forward primer and 1 µl of a reverse primer. For fungi 25 pmol of PET labelled ITS1f (5′-CTT GGT CAT TTA GAG GAA GTA A-3′) [24] and unlabelled ITS4 (5′-TCC TCC GCT TAT TGA TAT GC-3′) [25] were used. For bacteria 5 pmol of VIC labelled 1087r (5′ –CTC GTT GCG GGA CTT ACC CC 3′) [26] and unlabelled 63f (5′-AGG CCT AAC ACA TGC AAG TC-3′) [27] were used. Thermocycling consisted of an initial denaturation at 95°C for 3 min followed by 30 cycles of 95°C for 30 s, 55°C for 60 s, 72°C for 60 s. The final extension was at 72°C for 10 min. The PCR products were combined before being purified using a Qiagen PCR purification step so that only one purification, restriction endonuclease digestion and sample for TRFLP analysis was required. Purified DNA (approximately 250 ng) was digested with *Hha*I or *Msp*I for 4 h at 37°C and the reaction terminated by a further incubation at 95°C for 15 min. Aliquots (1 µl) of digested PCR products were mixed with 10 µl of HIDI formamide (Applied Biosystems™, Warrington, UK) and 0.15 µl of internal size standard LIZ 1200 (Applied Biosystems™, Warrington, UK) and then denatured for 5 min at 95°C. TRFLP analysis was carried out on an automated sequencer, ABI PRISM1 3130×l Genetic Analyzer on a 36 cm capillary array (Applied Biosystems™, Warrington, UK). Terminal restriction fragments (TRFs) generated by the sequencer were analysed using GeneMarker 1.60 (SoftGenetics LLC®, USA). To avoid detection of primers and undigested PCR products, TRFs smaller than 70 bp or larger than 500 bp (fungi) or 900 bp (bacteria) were excluded from further analysis. The relative abundance of TRFs was determined by calculating the percentage height of each peak in relation to the total peak height of all peaks within one sample. This relative abundance data was used for statistical analysis.

### Cloning and Sequencing

Unlabelled primers were used to amplify fungal or bacterial rhizosphere DNA from pooled samples of four replicate plots of continuous OSR. PCR products were cloned using the QIAGEN PCR cloning plus kit (Qiagen, Crawley, UK). Plasmid DNA from 96 colonies underwent Templiphi™ amplification to provide the sequencing template (GE Healthcare Life Sciences, UK) [28]. Sequencing was carried out using the vector targeted PCR primers M13 forward (−40) and M13 reverse (Qiagen, Crawley, UK) on an automated sequencer (ABI PRISM1 3130×l Genetic Analyzer) using the BigDye® version 3.1 sequencing chemistry. Sequences were assembled and trimmed to the primer sites using the DNAstar, Inc. software suite. *In silico* restriction cut sites were then determined. The sequences were compared with the Genbank database using the BLASTN program [29] and the ribosomal database project (RDP) [30] for phylogenetic comparison. The sequences obtained in this study are available in GenBank under accession numbers GenBank accession JF432891−JF433024.

### Identification of TRFs Using the Clone Libraries

The clone libraries were used to identify TRFs which were contributing towards the differences in microbial community structure. *In silico* digests of the clone library data were used to identify TRFs. TRFs may be up to 7 bp different to their *in silico* digest site [31], [32], so each DNA clone of interest was digested with the restriction enzyme used for TRFLP analysis (*Hha*I) to confirm TRF sizes. Each TRF was further validated by determining the presence of the predicted size of TRF using a second restriction enzyme (*Msp*I). Identification was only possible for TRFs of high abundance or well-spaced TRFs. Although the presence of another sequence with a restriction site in the same place as an unrelated sequence is always a possibility and can never be completely ruled out, there were no other unrelated sequences in the clone libraries with the same restriction sites for both restriction enzymes.

### Statistical Analysis

For statistical analysis, the TRFs produced using the restriction enzyme *Hha*I were used. This restriction enzyme was found to give the largest number of peaks which were also relatively evenly spaced. TRFLP community profiles were expressed in relative abundance and analysed for resemblance using analysis of similarity (ANOSIM) and non-metric multidimensional scaling (non-metric MDS) (PRIMER, version 6, Primer-E) [33]. ANOSIM reports the level of dissimilarity between sample groups (global *R*) and the associated level of significance (*P*). *R* is scaled to be within the range +1 to −1. Positive *R* values indicate that samples are more dissimilar between groups than within groups. *R* values close to zero occur if the high and low similarities are perfectly mixed and bear no relationship to the group. Negative *R* values indicate that dissimilarities within groups are greater than dissimilarities between groups [33]. Significance values were obtained by permutation tests. The relative contribution (%) of each TRF to the similarity matrix structure was assessed using SIMPER (Similarity Percentages - species contributions) [33]. TRFs which had a relative abundance of over 0.5% and real-time PCR copy numbers were tested by ANOVA which was used to compare their abundance across rotations.

### Real-time PCR

Unique DNA sequences within the ITS region were identified for *Olpidium brassicae* (Ob) and *Pyrenochaeta* sp. (Py) using the fungal clone library and the BLAST database (NCBI). Real-time PCR primers were then designed within these regions using Primer Express 2.0 (Applied Biosystems); ObF (5′-TCT CCT CGT TGG GAA GAC TTG T-3′) and ObR (5′-GAG CTT GAA TTT TTA AGT TCG TCG TT-3′); PyF (5′-CCG CCG GTT GGA CAC TAT AA-3′) and PyR (5′-TCG ATG CCA GAA CCA AGA GAT-3′). To confirm the primer specificity they were used to amplify DNA from rhizosphere samples which had been shown by TRFLP to contain approximately 1% of the total fungal population of either *O. brassicae* or *Pyrenochaeta* sp. The PCR was carried out using the real-time PCR conditions (below) and the products were cloned and sequenced. All of the clones (48) had the correct sequences with no other organisms being amplified.

Cloned *O. brassicae* or *Pyrenochaeta* sp. ITS DNA was quantified using the NanoDrop® ND-1000 spectrophotometer (NanoDrop Technologies). A standard curve of a fivefold dilution of each was made from 32 pg ml^−1^ down to 4.1×10^−3^ pg ml^−1^. Total rhizosphere or bulk soil DNA (1 ng) was also quantified using the NanoDrop® ND-1000 spectrophotometer (NanoDrop Technologies) and used in the real-time PCR using the SYBR Green PCR master mix (Applied Biosystems). This gave target DNA concentrations within the standard curve. Each reaction was set up in triplicate in a 384-wellplate with the following components: 2×SYBR Green PCR mastermix (10 µl), 1 mM forward primer, 1 mM reverse primer, standard curve DNA or 1 ng total sample DNA, 400 µg ml^−1^ non-acetylated BSA and water added to 20 µl. Real-time PCR was carried out using the ABI Prism 7900 HT sequence detection system (Applied Biosystems) with standard Taqman cycling conditions (40 cycles of 95°C for 15 s followed by 1 min at 60°C). An average of the triplicate results was taken. The quantities of DNA obtained were converted to copy numbers of target DNA/µg total sample DNA.

### Isolation of Cultures and Inoculum Preparation

A culture of *O. brassicae* was established through isolation of zoosporangia from infected plant material collected from the field site, and stock cultures were subsequently maintained on oilseed rape seedlings grown in a soilless substrate (sand:terragreen; 50∶50 v:v) in the glasshouse. Zoospores were amplified in OSR plants, extracted in GS (0.05 M glycine, 1% sucrose), counted and diluted to provide a range of doses in the glasshouse experiments. Seedlings were grown for 10 days before inoculation with *O. brassicae*, and at this time 1 ml of the required zoospore dilution was pipetted onto the base of each seedling.

*Pyrenochaeta* sp. was isolated onto tap water agar from a root lesion found on an OSR plant collected from the field site. It was subsequently grown on an agar medium containing OSR leaf extracts. The pure culture of *Pyrenochaeta* sp. would not sporulate in the laboratory and therefore mycelium was used as inoculum for the glasshouse experiments. Mycelium was grown on autoclaved wheat grain for 7 weeks before being macerated in water. Half of the resulting liquid inoculum was autoclaved for use as negative controls. Dilutions of the liquid inoculum were used to provide a range of doses in the glasshouse experiments, and were incorporated into the growing medium at the time of planting.

### Glasshouse Experiments

*Brassica oleracea* was used as a model Brassica for seed yield experiments, as it is a rapid-cycling Brassica suited to glasshouse conditions. The effect of *O. brassicae* and *Pyrenochaeta* sp. on early growth of OSR and *B. oleracea,* and seed yield of *B. oleracea,* was assessed in a soilless substrate using a sand-terragreen mix as a growing medium. This system allowed the candidate microorganisms to be tested in isolation, without the presence of other soil microorganisms.

### Early Growth Experiments

OSR (*Brassica napus* cv. Winner) and *Brassica oleracea* (DHSL150) plants were inoculated independently with the selected fungi in a range of doses. The three doses of *Olpidium brassicae* were ‘High’ = 1×10^7^ zoospores seedling^−1^, ‘Medium’ = 4×10^4^ zoospores seedling^−1^ and ‘Low’ = 2.5×10^2^ zoospores seedling^−1^. A control was also set up using GS alone. The three doses of *Pyrenochaeta* sp. were ‘High’ = 4.7×10^4^ (±1.0×10^4^) cfu g^−1^ substrate, ‘Medium’ = 4.7×10^2^ (±1.0×10^2^) cfu g^−1^ substrate and ‘Low’ = 4.7 (±1.0) cfu g^−1^ substrate, each of which had a corresponding autoclaved control. Seedlings were grown for 6–7 weeks before top growth and root biomass were recorded, and root material (0.4 g) was taken for DNA extraction and analysis of the inoculated fungi using real-time PCR. For each replicate, two pots per sample were used with 3 plants in each pot to maximise root material for DNA analysis without impacting on overcrowding in the pots. Four complete replicates were set up.

### Seed Yield Experiments

*B. oleracea* plants were again inoculated independently with the selected fungi in a range of doses and were grown to seed set. The three doses of *Olpidium brassicae* were ‘High’ = 1×10^7^ zoospores seedling^−1^, ‘Medium’ = 4×10^4^ zoospores seedling^−1^ and ‘Low’ = 2.5×10^2^ zoospores seedling^−1^. A control was also set up using GS alone. The three doses of *Pyrenochaeta* sp. were ‘High’ = 4.9×10^4^ (±0.97×10^4^) cfu g^−1^ substrate, ‘Medium’ = 4.9×10^2^ (±0.97×10^2^) cfu g^−1^ substrate and ‘Low’ = 4.9 (±0.97) cfu g^−1^ substrate, each of which had a corresponding autoclaved control. At harvest, assessments were made on various components of yield, including number of branches, number of pods, number of seed-containing pods, and number and weight of normal (smooth and spherical) or deformed (flattened and/or wrinkled) seeds. Rhizosphere samples (0.4 g) were also taken at harvest for DNA extraction and quantification of inoculated fungi using real-time PCR. For each replicate, one pot per treatment was used with a single plant per pot, and ten complete replicates were set up.

## Results

### Fungal Community and TRF Identification

**Figure 1:**
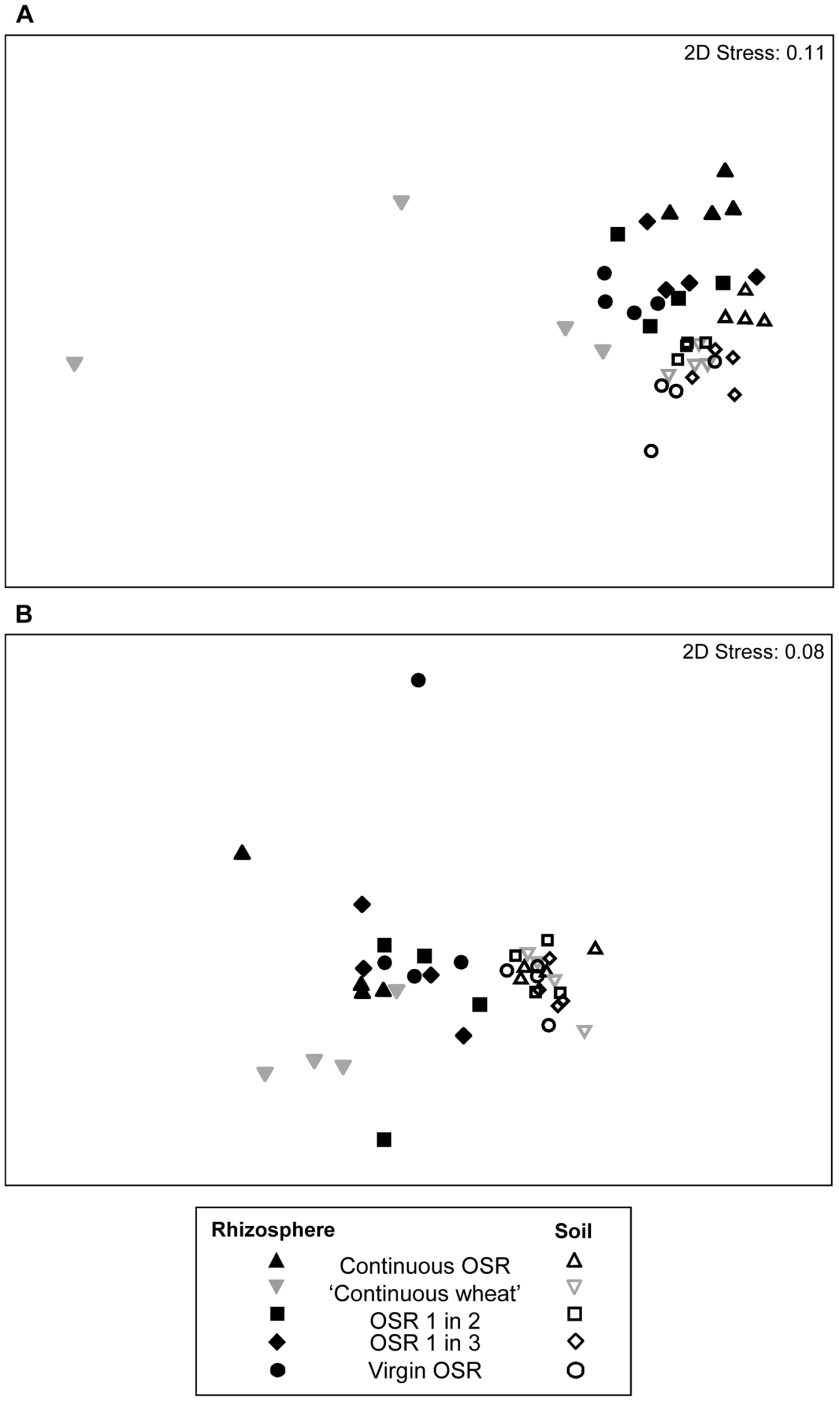
Non-metric multi-dimensional scaling plots represent rhizosphere (solid) and bulk soil (open) DNA profiles generated using primers for: (a) fungi or (b) bacteria, obtained from different rotations of rape (black) and wheat (grey).

**Figure 2:**
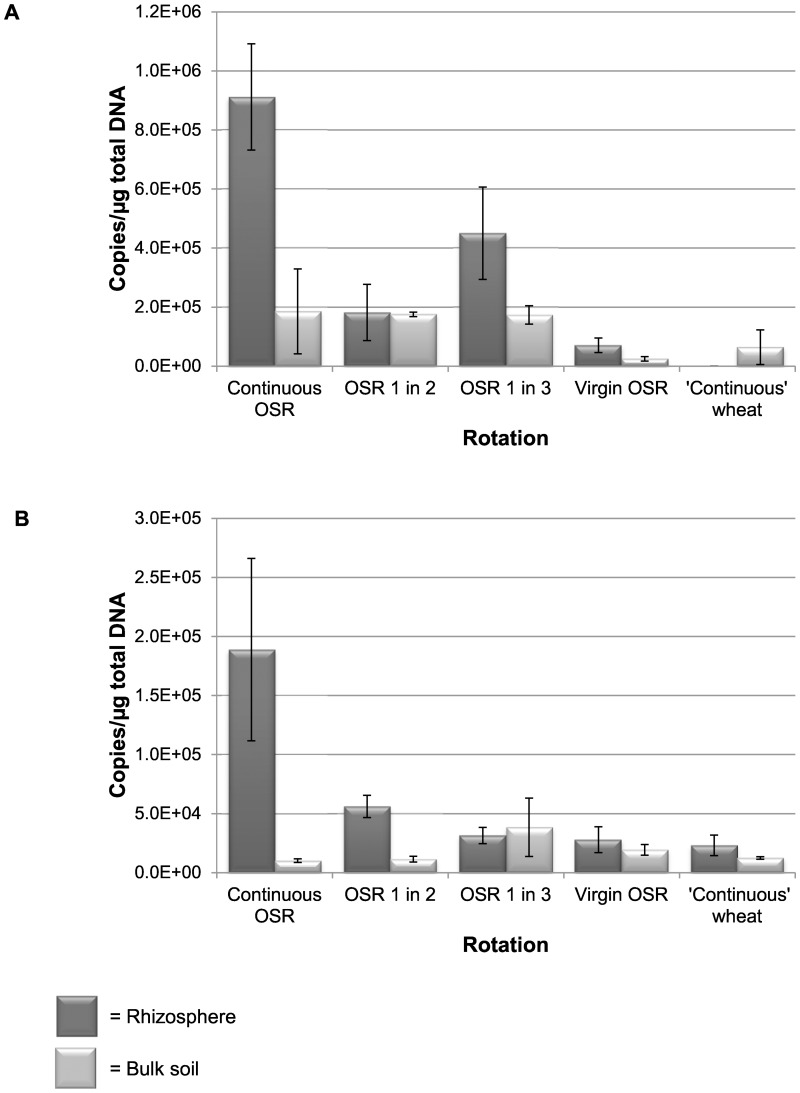
Absolute quantification using specific quantitative PCR primers to (a) *Olpidium brassicae* and (b) *Pyrenochaeta* sp. in different rotations of OSR and wheat (see **Table 1** for rotation explanation). Error bars are ± standard errors of the mean for the four replicate plots.

Non-metric MDS with ANOSIM analysis of the TRFLP data showed that rotation had a significant effect on the fungal community of the rhizosphere and of the bulk soil (Fig. 1a, Table 2). Within the OSR rotations, the continuous OSR and virgin OSR communities were the most diverse with the other OSR rotations (OSR grown every second or third year) containing communities between the two. The continuous OSR (rhizosphere and bulk soil) had significantly different fungal communities to all other rotations (Fig. 1a, Table 2). Using SIMPER analysis, the TRFs that contributed most towards the differences between the rhizosphere of continuous OSR and OSR grown after wheat were 284 bp and 98 bp which both had a higher relative abundance in the continuous OSR rhizosphere. These TRFs were identified using clone libraries. The TRF at 284 bp was identified as *Olpidium brassicae* as it had 99%–100% identity to *O. brassicae* isolated from cabbage roots (Table S1). Real-time PCR showed that there was significantly more *O. brassicae* in the continuous OSR rhizosphere than all other rotations (p = 0.001; Fig. 2a). There was a low amount of *O. brassicae* in the rhizosphere of virgin OSR and intermediate levels in the rotations where OSR was grown every second or third year. *Olpidium brassicae* was not found in the rhizosphere of wheat and low levels of *O. brassicae* were detected in the bulk soil samples.

**Table 2:** ANOSIM of fungal and bacterial communities.

Community	Soil type	Treatments compared	P	R
Fungi		Rhizosphere v bulk soil	**0.001**	**0.411**
	Rhizosphere	OSR Rotations	**0.002**	**0.401**
		Crop (OSR v Wheat)	**0.001**	**0.702**
		Continuous OSR v OSR grown after wheat	**0.002**	**0.554**
		Continuous OSR v Virgin OSR	**0.029**	**0.917**
	Bulk soil	OSR Rotations	**0.001**	**0.432**
		Crop (OSR v Wheat)	0.975	−0.284
		Continuous OSR v OSR grown after wheat	**0.008**	**0.454**
		Continuous OSR v Virgin OSR	**0.029**	**0.792**
Bacteria		Rhizosphere v bulk soil	**0.001**	**0.690**
	Rhizosphere	OSR Rotations	0.734	−0.041
		Crop (OSR v Wheat)	0.138	0.204
		Continuous OSR v OSR grown after wheat	0.374	0.047
		Continuous OSR v Virgin OSR	0.057	0.167
	Bulk soil	OSR Rotations	0.845	−0.104
		Crop (OSR v Wheat)	0.362	0.052
		Continuous OSR v OSR grown after wheat	0.178	0.157
		Continuous OSR v Virgin OSR	0.657	−0.073

P-values showing significance of the effects of factors (rotation and crop) on the microbial communities of the rhizosphere and bulk soil for fungi and bacteria. R values close to zero indicate most similarity. Values in bold highlight significant differences (p = <0.05).

The TRF at 98 bp was identified as *Pyrenochaeta* sp. as it had 100% identity to a *Pyrenochaeta* sp. isolated from the grass species *Ammophila arenaria* (Table S1) as well as a match at 95% DNA identity to *Pyrenochaeta lycopersici* (causal organism of tomato corky root). Real-time PCR showed that there was significantly more *Pyrenochaeta* sp. in continuous OSR rhizosphere than in all other treatments (p = 0.012; Fig. 2b). Very little *Pyrenochaeta* sp. was detected in any of the bulk soil samples and there were no significant differences between rotations.

Using SIMPER analysis, the TRFs that each contributed most towards the differences between the bulk soil of continuous OSR and OSR grown after wheat were 124/125 bp which was identified as *Gibellulopsis nigrescens* (previously named *Verticillium nigrescens* [34]) (Table S1) and 284 bp (*O. brassicae*) which both had a significantly higher relative abundance in the bulk soil of continuous OSR compared to OSR grown after wheat (124/125 bp, p = 0.001; 284 bp, p = 0.042). A number of other less abundant TRFs were also found to have a significantly higher relative abundance in the continuous OSR bulk soil to other rotations (data not shown).

Crop type was shown to have a significant effect on the fungal community in the rhizosphere, but not on the fungal community in the bulk soil (Fig. 1a, Table 2). In the bulk soil there were larger differences within OSR rotations than between OSR rotations and wheat rotations (Fig. 1a, Table 2). This was due to the higher relative abundance of TRFs 124/125 bp (*G. nigrescens*) and 284 bp (*O. brassicae*) in the continuous OSR bulk soil relative to all other rotations. There was also a significant difference in the fungal community of the rhizosphere compared with the bulk soil (Fig. 1a, Table 2). Using SIMPER analysis, the TRFs that contributed most towards the differences between the rhizosphere and bulk soil communities were TRF 284 bp (*O. brassicae*) and TRF 341 bp (identified as *Tetracladium* sp.) (Table S1) which were both found in higher relative abundance in the rhizosphere, and TRF 124/125 bp (*G. nigrescens*) which was found in higher relative abundance in the bulk soil.

### Bacterial Community and TRF Identification

Non-metric MDS with ANOSIM analysis of the TRFLP data showed that rotation and crop type did not have a significant effect on the overall bacterial community in the rhizosphere or the bulk soil (Fig. 1b, Table 2).

There was a significant difference in the bacterial community of the rhizosphere compared with the bulk soil (Fig. 1b, Table 2). The TRFs that each contributed most towards the differences between the rhizosphere and bulk soil were TRFs 523 bp (Burkholderiales) and 245 bp (identified as *Pseudomonas fluorescens*), which had a higher relative abundance in the rhizosphere, and TRFs 338 bp and 721 bp (both identified as members of the phylum Acidobacteria Gp6), which had a higher relative abundance in the bulk soil (Table S1).

### Glasshouse Experiments Using Model System

#### Olpidium brassicae: Early growth experiment

**Figure 3:**
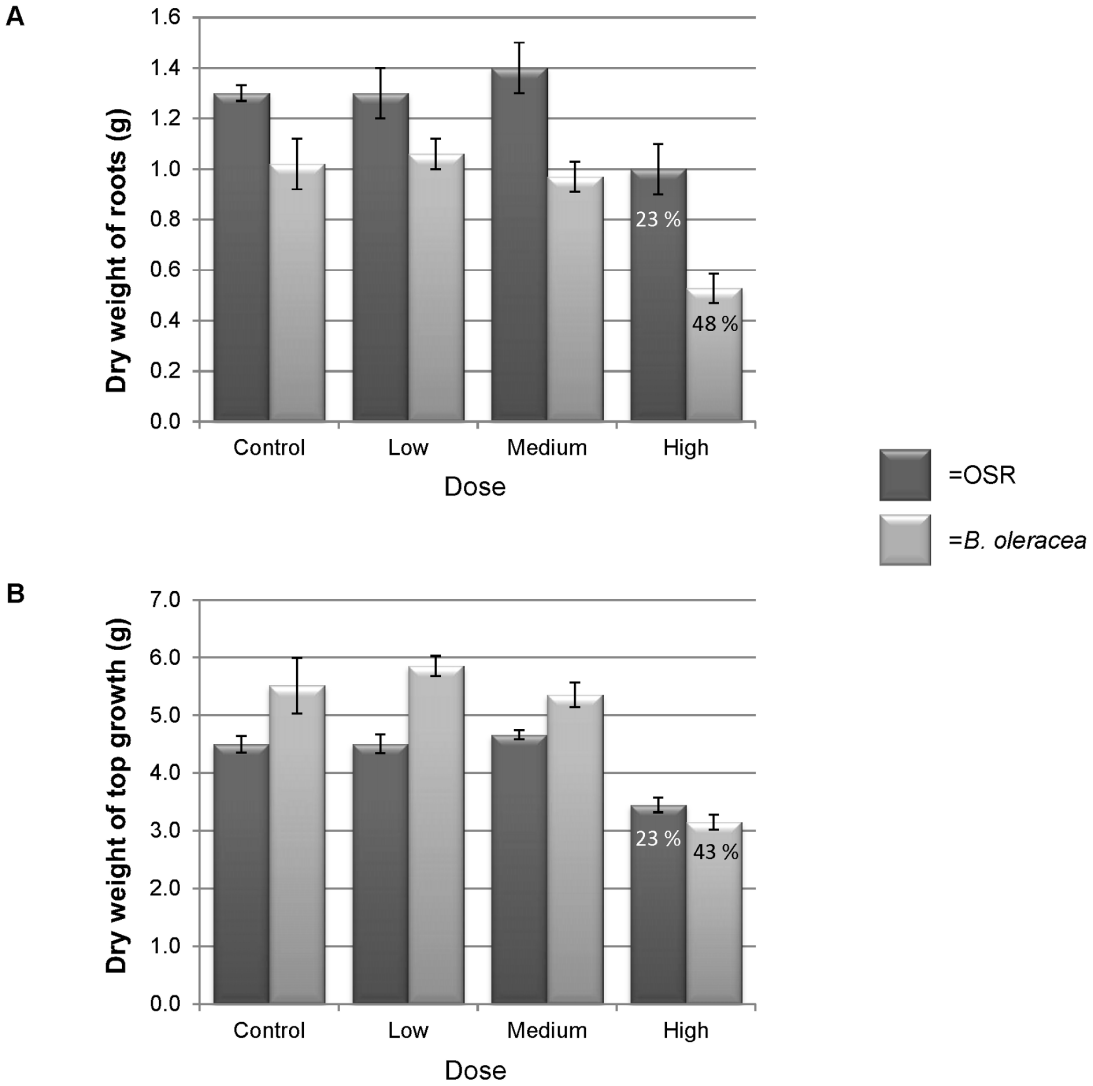
Dry weights of roots and top growth of oilseed rape and *Brassica oleracea* plants grown in a soilless substrate for seven weeks inoculated with zoospores (zs) of *Olpidium brassicae.* (a) roots (b) top growth. Control = 0 zs seedling^−1^; Low = 2.5×10^2^ zs seedling^−1^; Medium = 5×10^4^ zs seedling^−1^; High = 1×10^7^ zs seedling^−1^. Percentages in the ‘High’ columns indicate yield reduction compared with the control. Error bars are ± standard errors of the mean for the four replicates.

After 6–7 weeks growth, a significant reduction in top growth and root biomass occurred in plants inoculated with the High dose, for both OSR and *B. oleracea* (*P*≤0.01; Fig. 3). Brown discolouration of the roots was also observed in the High dose plants. There was no difference in top growth or root weight between the control plants and the Low or Medium dose treatments.

#### Olpidium brassicae: Seed yield experiment

**Figure 4:**
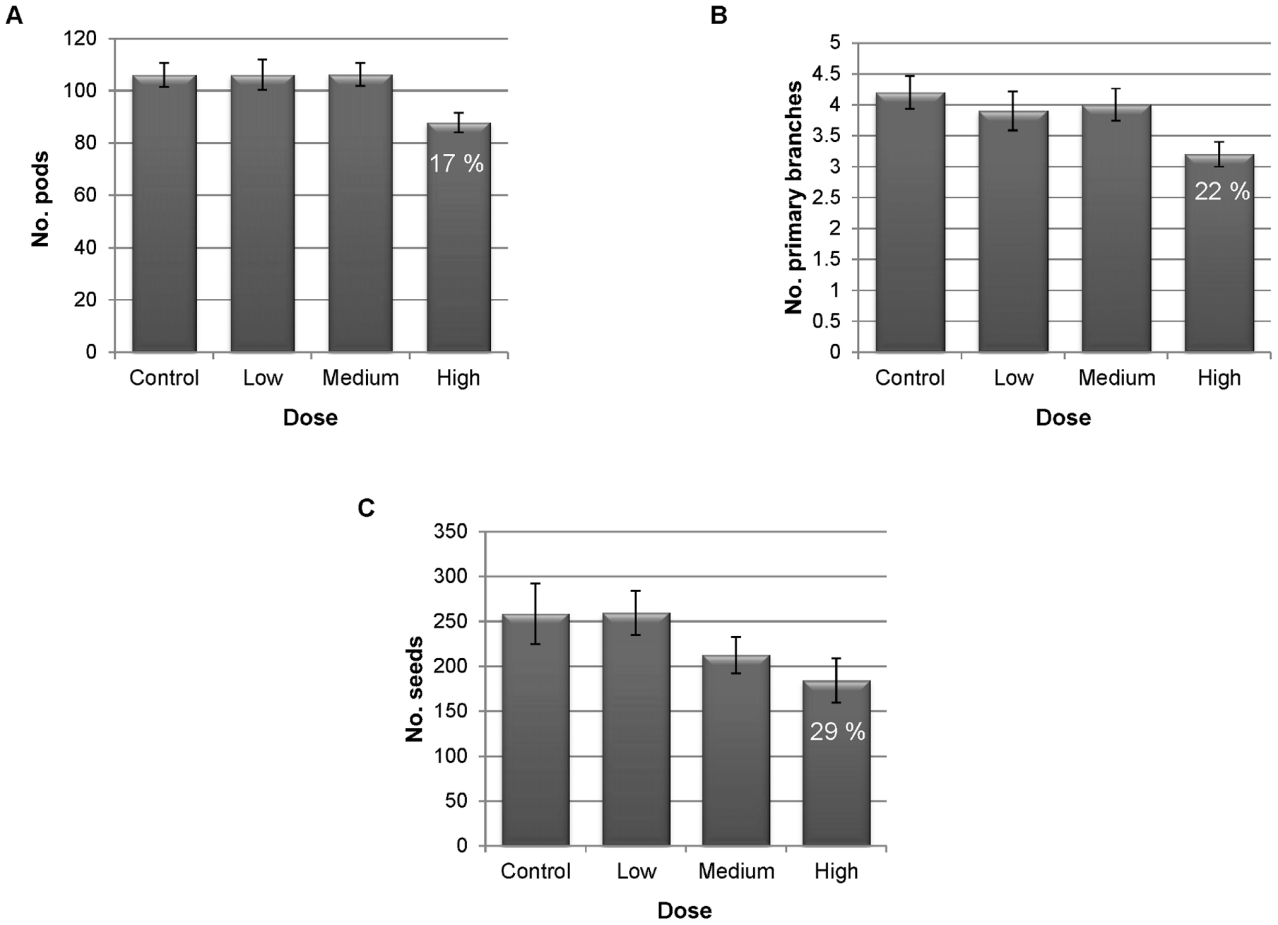
Seed yield components of *Brassica oleracea* plants grown in a soilless substrate for four months after inoculation with zoospores (zs) of *Olpidium brassicae.* (a) pods (b) primary branches (c) seeds. Control = 0 zs seedling^−1^; Low = 2.5×10^2^ zs seedling^−1^; Medium = 5×10^4^ zs seedling^−1^; High = 1×10^7^ zs seedling^−1^. Error bars are ± standard errors of the mean for the ten replicates.

At harvest, there was a significant reduction in total number of pods produced in the plants inoculated with the High dose of *O. brassicae* (p = 0.017; Fig. 4a). This was found to be predominantly due to a reduced potential for pod production in this treatment as these plants also produced fewer primary branches than the uninoculated control (Fig. 4b). This ultimately resulted in a reduction in the number of seeds produced (p = 0.015; Fig. 4c). There was no difference between the control plants and the Low or Medium dose treatments.

#### Pyrenochaeta sp.: Early growth experiment

**Figure 5:**
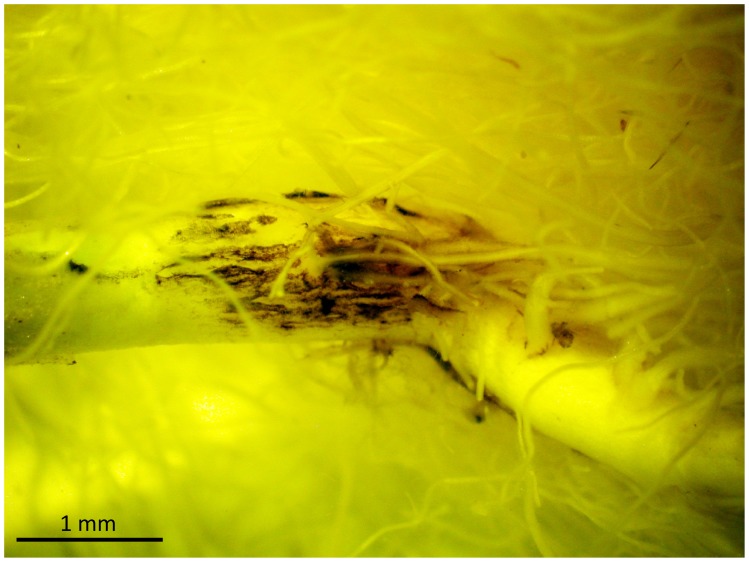
*B. oleracea* root displaying a root lesion after inoculation with *Pyrenochaeta* sp.

After 6–7 weeks growth, there was no effect of *Pyrenochaeta* sp. on the top growth or root biomass at any dose, although the High dose autoclaved inoculum was found to have a phytotoxic effect on *B. oleracea*. Medium and high doses showed brown discolouration of the roots compared to the autoclaved controls. Lesions were observed on the roots of inoculated plants (Fig. 5).

#### Pyrenochaeta sp.: Seed yield experiment

**Figure 6:**
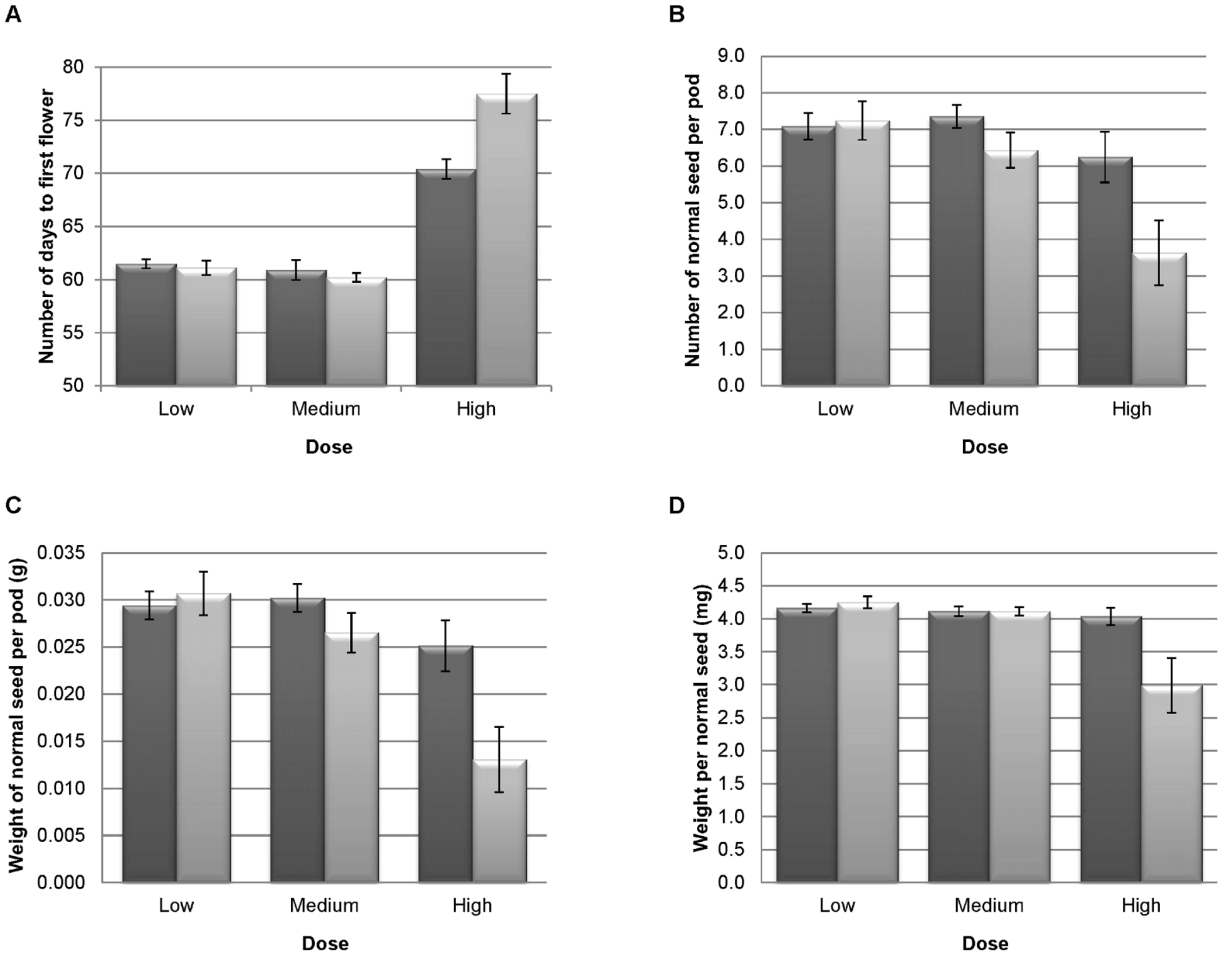
Seed yield components of *Brassica oleracea* plants grown in a soilless substrate for four months containing *Pyrenochaeta* sp. mycelium. (a) days to first flower (b) no. normal seeds/pod (c) weight of normal seeds/pod (d) weight per normal seed. Light grey = live substrate; Low = 4.9 (±0.97) cfu g^−1^ substrate, Medium = 4.9×10^2^ (±0.97×10^2^) cfu g^−1^ substrate, High = 4.9×10^4^ (±0.97×10^4^) cfu g^−1^ substrate. Dark grey = autoclaved substrate control. Error bars are ± standard errors of the mean for the ten replicate plants.

Plants inoculated with a High dose of *Pyrenochaeta* sp. had a delay in time to first flower (7 days) compared to the equivalent control (Fig. 6a). Subsequent analyses of the seed yield components revealed that the High dose had a negative impact on seed quantity and quality. The High dose resulted in significantly fewer normal seeds per pod compared to its control (p = 0.044; Fig. 6b), and a correspondingly greater number of deformed seed in total compared to the autoclaved control (p = 0.016). As a result of the reduction in number of normal seeds at the High dose, the seed weight per pod was also significantly less compared to the autoclaved control (p = 0.019; Fig. 6c). Not only were fewer normal seeds produced per pod, but those that were produced also weighed less per seed (p = 0.009; Fig. 6d). The Medium dose resulted in significantly fewer total number of seeds (including all normal and deformed seeds) compared to its autoclaved control (p = 0.028). However, the individual component analysis did not reveal further significant differences in number and weight of seed per pod for the Medium dose. There were no significant effects of the Low dose of *Pyrenochaeta* sp. on any yield component assessed.

#### Real time PCR

Real-time PCR confirmed the presence of either *O. brassicae* or *Pyrenochaeta* sp. in the roots of the inoculated samples, which was always highest in the ‘High’ dose inoculated plants. The fungi were not detected in the control samples (data not shown).

## Discussion

This study has demonstrated that there is a strong influence of OSR rotation on microbial community structure and results from the field trial support reports that OSR cropping frequency has an impact on yield. Although shifts in microbial populations have previously been identified associated with rotation, consequences for crop growth have not been investigated [16]–[21]. In this study, we have taken further steps to identify the microbes responsible for the shifts in population, resulting in their isolation and functional studies.

Crop rotation was shown to have a significant effect on the fungal community. This was due predominantly to the increase in abundance of specific fungi in continuously cropped OSR, which suggests that the differences in rotation are due to enrichment of these fungi when OSR is planted. Greatest community divergence was observed between the fungal rhizosphere communities of continuous OSR and virgin OSR, with the other OSR rotations consisting of intermediate communities. Two microorganisms that became more prominent in the rhizosphere with frequent cropping of OSR were identified and selected for further investigation.

The first fungus, which showed the largest increase in abundance in the rhizosphere of continuous OSR compared with virgin OSR, was identified as *Olpidium brassicae* which is a soil-borne fungus within the phylum Chytridiomycota. *Olpidium brassicae* s.l. is a root-infecting obligate plant parasite, which is widespread in temperate regions but is reported to seldom induce obvious symptoms in host plants [35]–[37]. Two distinct species of *O. brassicae* s.l. have been reported; one that infects brassicas and one that infects non-brassicas, and phylogenetic analysis showed that the species are clearly distinct [35], [38]. The non-brassica-infecting *O. brassicae* s.l. has been tentatively renamed *O. virulentus*; this is the species known to transmit destructive plant viruses [35]. The brassica-infecting *O. brassicae* s.l. species has retained the name *O. brassicae.* To date, no viruses have been identified that are associated with *O. brassicae*. Hosts of *O. virulentus* include lettuce, cucumber, tobacco and onion; whereas *O. brassicae* is exclusive to the roots of brassicas, including cabbage, brussel sprouts, broccoli and oilseed rape [38], [39]. In this study, it was demonstrated that in a model system, at high doses, *O. brassicae* significantly reduced both top growth and root biomass of OSR and *B. oleracea* seedlings and impacted on *B. oleracea* seed yield by reducing branching and consequently total pod and seed production.

The second fungus identified was *Pyrenochaeta* sp.; an ascomycete which has a 95% DNA identity to *Pyrenochaeta lycopersici;* the causal agent of tomato corky root. Corky root is a severe disease that affects tomato crops worldwide [40]. Visible symptoms of tomato corky root are brown lesions in the roots and swelling of root epidermis with subsequent cracking into a corky texture [40]. The progressive damage to the root system leads to severe losses in fruit yield. Tomato yield reductions of up to 75% have been observed in European greenhouses [41]. In this study, it was demonstrated that in a model system, *Pyrenochaeta* sp. resulted in the development of root lesions, which may correspond to the root lesions found on tomato plants during *P. lycopersici* infection. In high doses, *Pyrenochaeta* sp. also delayed flowering and reduced seed weight, quality and quantity per pod of *B. oleracea*.

Overall, results indicate that the two fungi introduced at high doses, did impact on the seed yield production of the model species *B. oleracea*. Interestingly, the effects on the plants were different; *O. brassicae* reduced the number of branches, thus reducing the potential for seed production, whereas *Pyrenochaeta* sp. affected seed quantity and quality per pod. Although these results are from a model system, they highlight different mechanisms for yield reduction. Whilst the model system (using a soilless substrate under glasshouse conditions) was useful in testing the effects of individual fungal isolates on brassica growth and yield, it is now important to determine the impact of these fungi in soil, where interactions with other microorganisms will occur. The entry points and lesions created by *O. brassicae* and *Pyrenochaeta* sp. respectively, are likely to allow secondary infections which may also contribute to symptoms of yield decline. During this study, other potential pathogens were identified. For example, in the bulk soil of continuously cropped OSR, *Gibellulopsis* (formerly *Verticillium*) *nigrescens* had a greater relative abundance in continuously cropped OSR compared with virgin OSR. The relative importance of other potential pathogens needs to be established as it is likely that the observed yield decline is due to a combination of interacting factors involving a pathogen complex. Furthermore the interactions including any synergistic or antagonistic effects between the pathogens need to be determined.

Yield decline appears to be a nationwide and likely a world-wide phenomenon [2]. It is therefore important to determine whether these potential pathogens are found in field sites other than the site used in this study. A preliminary survey has shown that *Olpidium brassicae* is wide-spread and has been found to be associated with both winter and spring OSR, at different field sites across the UK, with higher levels found in the majority of intensively cropped OSR field sites (data not shown).

This study has identified two brassica pathogens from the field trial which contribute to yield decline in the model system; *Olpidium brassicae,* which has generally been overlooked as a brassica pathogen, and *Pyrenochaeta* sp., which has not previously been identified as a brassica pathogen. Future research is required to determine the effects and interactions of these fungi under field conditions and to investigate other identified fungi as well as other microorganisms. It is also important to understand how cultural and environmental factors influence these microorganisms in order for sustainable agricultural systems to be developed.

## Supporting Information

Figure S1 **Yield data from plots within different rotations of OSR (in rotation with wheat), from the fourth year of the field trial (2007).** Error bars are ± standard errors.
(TIF)

Table S1 **Identification of TRFs using the continuous OSR rhizosphere clone library.** NCBI BLAST and Ribosomal Database Project (RDP) (at 80% confidence) were used to classify fungal (a) and bacterial (b) TRFs, respectively. TRF sizes and equivalent cut sites in the clones are shown using *Hha*I and *Msp*I. The accession number of the closest match of the consensus of the clones is shown for the fungal clones. * = An overlapping restriction site occurs resulting in a double peak.
(DOCX)
