# Supplementary figures and images for: Impact of Shortened Crop Rotation of Oilseed Rape on Soil and Rhizosphere Microbial Diversity in Relation to Yield Decline
Source: PLoS One. 2013 Apr 1;8(4):e59859. doi: 10.1371/journal.pone.0059859 (PMC3613410; doi:10.1371/journal.pone.0059859)

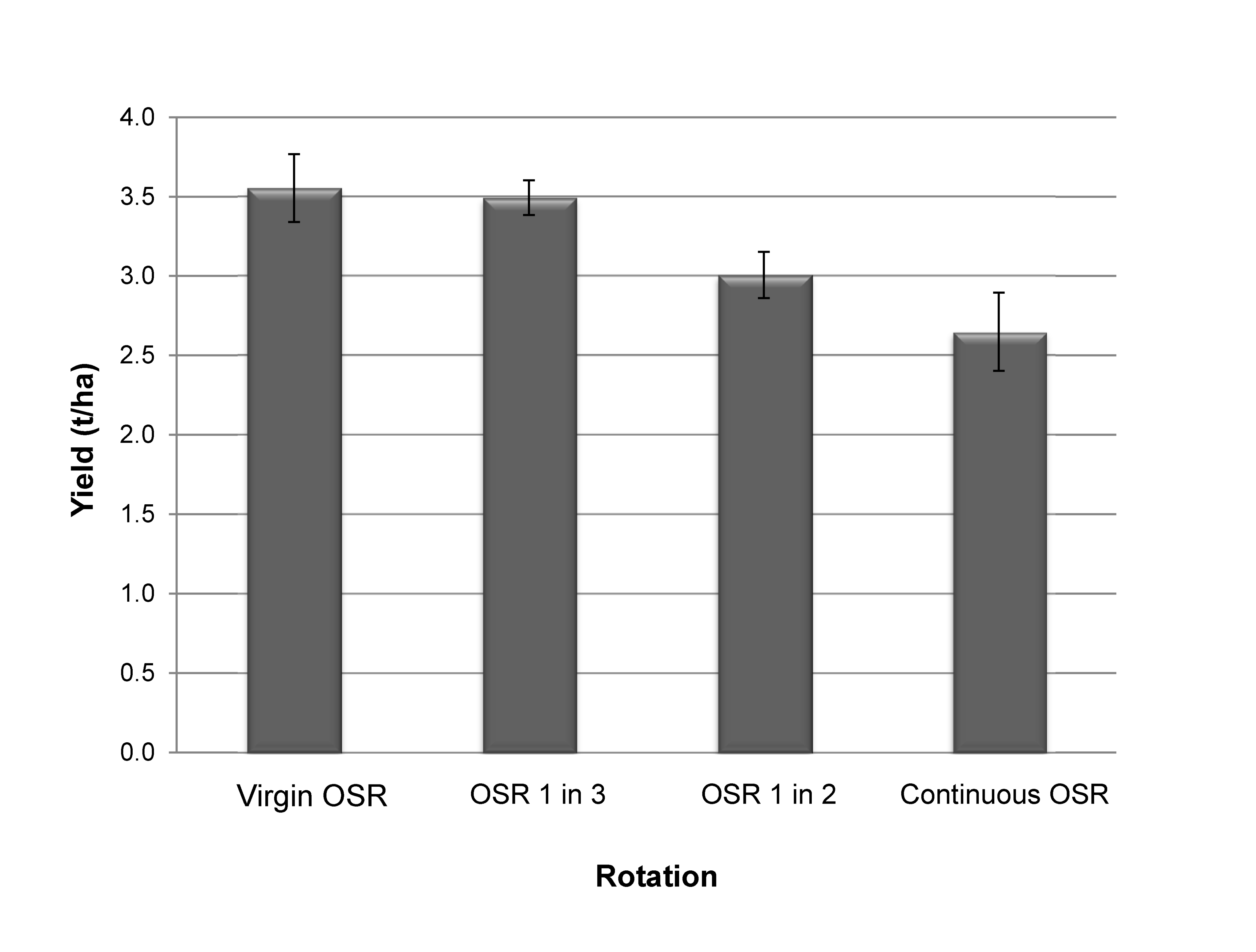

Supplement: Figure S1 — Yield data from plots within different rotations of OSR (in rotation with wheat), from the fourth year of the field trial (2007). Error bars are ± standard errors. (TIF) [file pone.0059859.s001.tif]
