# Supplementary material for: Impact of Shortened Crop Rotation of Oilseed Rape on Soil and Rhizosphere Microbial Diversity in Relation to Yield Decline
Source: PLoS One. 2013 Apr 1;8(4):e59859. doi: 10.1371/journal.pone.0059859 (PMC3613410; doi:10.1371/journal.pone.0059859)
Supplement: Table S1 — Identification of TRFs using the continuous OSR rhizosphere clone library. NCBI BLAST and Ribosomal Database Project (RDP) (at 80% confidence) were used to classify fungal (a) and bacterial (b) TRFs, respectively. TRF sizes and equivalent cut sites in the clones are shown using HhaI and MspI. The accession number of the closest match of the consensus of the clones is shown for the fungal clones. * = An overlapping restriction site occurs resulting in a double peak. (DOCX) [file pone.0059859.s002.docx]

Table S1. Identification of TRFs using the continuous OSR rhizosphere clone library.

| **A** | **% of clone library** | **Accession numbers** | ***Hha*I TRF (bp)** | ***Hha*I site**  **(bp)** | ***Msp*I TRF (bp)** | ***Msp*I site (bp)** | **Top NCBI Blast hit / Accession no.** | **ID/ range (bp)** |
| --- | --- | --- | --- | --- | --- | --- | --- | --- |
|  | 11.9 | JF432964; JF432970; JF432976 | 124/125* | 125/127* | 130 | 132 | *Gibellulopsis nigrescens* / AM922222 | 99 %/ 505 |
|  | 9.5 | JF432979; JF432982; JF432988  JF432998; JF433001; JF433008  JF433012 | 284 | 286 | 290 | 291 | *Olpidium brassicae* / AB205209 | 99-100 %/ 600 |
|  | 7.1 | JF432972; JF432989; JF433000  JF433002; JF433021 | 123 | 124 | 135 | 141 | *Plectosphaerella cucumerina* / L36640 | 98-100 %/ 573 |
|  | 7.1 | JF432973; JF433004 | 325 | 330 | 480 | 490 | *Trichothecium* sp. / EU754905 | 99 %/ 546 |
|  | 2.4 | JF432995 | 98 | 99 | 83 | 84 | *Pyrenochaeta* sp. / AM921726 | 100 %/ 506 |
|  | 2.4 | JF432975; JF432987 | 341 | 344 | - | - | *Tetracladium furcatum* / EU883432 | 100%/ 592 |
|  | 2.4 | JF432993; JF433006 | 299 | 301 | 312 | 314 | *Trichosporon* sp. / FJ439589 | 100 %/ 528 |
| **B** | **% of clone library** | **Accession numbers** | ***Hha*I TRF**  **(bp)** | ***Hha*I site (bp)** | ***Msp*I TRF (bp)** | ***Msp*I site (bp)** | **RDP classification** | |
|  | 7.0 | JF432908; JF432913; JF432920  JF432922 | 245 | 250 | 486 | 491 | *Pseudomonas* spp. | |
|  | 4.2 | JF432898; JF432926; JF432947 | 523 | 528 | 487 | 492 | Burkholderiales | |
|  | 2.8 | JF432903; JF432931 | 338 | 344 | 300 | 306 | Acidobacteria Gp 6 | |
|  | 1.4 | JF432934 | 721 | 727 | 300 | 306 | Acidobacteria Gp 6 | |

NCBI BLAST and Ribosomal Database Project (RDP) (at 80 % confidence) were used to classify fungal (a) and bacterial (b) TRFs, respectively. TRF sizes and equivalent cut sites in the clones are shown using *Hha*I and *Msp*I. The accession number of the closest match of the consensus of the clones is shown for the fungal clones. * = An overlapping restriction site occurs resulting in a double peak.
